# Occupational therapists’ perceived ability to facilitate reasonable accommodations in the workplace for employees with major depressive disorders

**DOI:** 10.4102/ajod.v15i0.1924

**Published:** 2026-06-13

**Authors:** Mpho S. Ramano, Tania Buys, Carla Kotzé

**Affiliations:** 1 Department of Occupational Therapy, Faculty of Health Sciences, University of Pretoria, Pretoria, South Africa; 2 Department of Geriatric Psychiatry, Faculty of Psychiatry, Weskoppies, Pretoria, South Africa

**Keywords:** employment equity, disability rights, equal opportunity, mental health stigma, workplace productivity, competencies, return-to-work

## Abstract

**Background:**

Employees with major depressive disorder who return to work after sick leave may exhibit residual symptoms and functional limitations, which might require reasonable accommodation. In accordance with disability legislation, these employees have the right to request reasonable accommodations, and the employer must consider these requests, allowing the employee to continue fulfilling work requirements. There is a lack of research concerning the occupational therapist’s role and successes in supporting reasonable accommodation for employees with major depressive disorders.

**Objectives:**

This article aims to explore and describe occupational therapists’ perceived ability to facilitate reasonable workplace accommodations for employees with major depressive disorders.

**Method:**

An exploratory descriptive design was utilised, in which seven participants were interviewed individually. The authors adopted a social constructivism paradigm to understand the world in which occupational therapists live and work. An inductive analysis was carried out to formulate the themes.

**Results:**

Two themes were identified from the participants’ views: (1) the dynamic process of reasonable accommodation and (2) the competencies of occupational therapists.

**Conclusion:**

Occupational therapists should lead the dynamic process of reasonable accommodation. To do this effectively, they must stay up to date, have the relevant skills and manage cases efficiently.

**Contribution:**

The dynamic process of reasonable accommodation outlined in this study provides a guideline for occupational therapists to follow when implementing such adjustments. Suggested reasonable accommodations for employees with major depressive disorder may serve as valuable resources for these professionals.

## Introduction

Worldwide, several laws support reasonable accommodation of employees with disabilities, including those diagnosed with major depressive disorders (MDD) (Buys 2015; *Employment Equity Act 55 1998*; *Labour Relation Act 66 1995*; Shamberg 2005). The UN Convention on the Rights of Persons with Disabilities (UN CRPD) (Ebuenyi et al. 2019) and the *South African Employment Equity Act* (EEA) *55 of 1998* in section one refer to reasonable accommodation as any necessary and appropriate modification or adjustment to a job or the working environment that enables employees with disabilities, including those with mental impairments, to have fair access to participate or advance in employment or to perform their essential job functions. Therefore, an employee with MDD has the right to request reasonable accommodations to participate fully and equally in the workplace, and the employer is obligated to consider these requests. The symptoms of MDD can affect the employee’s ability to perform work over time, which may lead to tension between the employee and the employer (Karjalainen & Ylhäinen 2021). The employer’s refusal to implement reasonable accommodations may be considered discrimination and impact the human dignity of the employee (Ebuenyi et al. 2019). The process of requesting and implementing reasonable accommodations should be carried out collaboratively between the employer, employee and occupational therapist. Joss (2011) and Ramano and Buys (2018) state that referral to an occupational therapist for a Functional Capacity Evaluation (FCE) is usually conducted for employees with MDD to aid in return-to-work decisions. Occupational therapists also provide intervention and a discharge plan for patients with MDD (AOTA 2020; Rocamora-Montenegro, Compañ-Gabucio & De La Hera 2021). Interventions for employees with MDD addressing cognitive and functional impairments include engagement and participation in meaningful activities, environment modification (reasonable accommodation) and cognitive rehabilitation (Alotaibi et al. 2024; Christie, Davys & Cook 2025; Rocamora-Montenegro et al. 2021). Occupational therapists, with their knowledge of MDD’s consequences on work functioning, legislation, a client-centred approach, negotiation skills and expert knowledge of work practices and accommodations, are well positioned to assist both employers and employees with MDD. During the reasonable accommodation process, occupational therapists need to employ an interactive process to foster effective cooperation and collaborative dialogue among the stakeholders, including the employee with MDD, supervisor, colleagues and union representatives (Bastien & Corbière 2019; Schreuer et al. 2009). Furthermore, occupational therapists apply problem-solving and clinical or professional reasoning skills (Alotaibi et al. 2024) to identify and implement suitable reasonable accommodations in the workplace. Facilitating successful reasonable accommodations involves the occupational therapist reducing the impact of the disability on work performance, building a trusting communication relationship between the employee with MDD and the employer and considering precautionary measures to prevent undue hardship or a direct threat to the employer (Bastien & Corbière 2019; Schreuer et al. 2009). Additionally, the success of reasonable accommodations can help the employer retain employees and ensure equal opportunities for work productivity, professional and personal growth and economic stability (Schreuer et al. 2009). In South Africa, one in six individuals experiences MDD (Booysen, Mahe-Poyo & Grant 2021). A study by Stander et al. (2016) revealed that MDD significantly impacts work performance, leading to absenteeism and presenteeism and causing an annual loss of R3.6 billion in earnings. Residual symptoms associated with functional limitations still persist even after return to work of employees with MDD following sick leave (Bastien & Corbière 2019; Bender & Farvolden 2008; Corbière et al. 2017). Even during remission, cognitive deficits affecting social and occupational functioning can persist (Bender & Farvolden 2008; De Vries et al. 2015; Woo et al. 2016). Common cognitive impairments in remitted MDD include inattention, impaired executive functioning and diminished verbal memory (Corbière et al. 2017; Woo et al. 2016). These residual and functional limitations highlight the importance of reasonable accommodations and the benefits they bring to the workplace. In comparison to people living with physical disabilities, people living with mental health disabilities are generally not understood or fully acknowledged in the workplace compared to those with physical disabilities (Follmer, Miller & Beatty 2024; Thisted et al. 2020; Van Bortel et al. 2024). Employers do not always provide reasonable accommodations for employees with MDD. This often leads to negative impacts such as work-related anxiety and fear of returning to work, stemming from employers’ reluctance to reasonably accommodate employees with MDD. Additionally, the failure to implement these accommodations appears to contribute to employees’ unnecessary relapses into episodes of depression (Bastien & Corbière 2019). As a result, employees with MDD often feel anxious about returning to work, fearing they will be unable to cope with job demands due to cognitive impairments associated with MDD. The findings of McDowell and Fossey (2015) support these observations, indicating that employees with MDD are less likely to receive reasonable accommodations compared to individuals with physical disabilities, due to stigma, the indefinable nature of appropriate accommodations, reluctance to disclose their mental condition or difficulties in accommodating an episodic condition. Franzsen et al. (2023) further emphasise that employees with MDD face stigmatisation from both the managers and colleague. Edgelow et al. (2020) suggested that occupational therapists should engage in this area of work practice and assert their unique role in the assessment and implementation of reasonable accommodations. Van Biljon, Rabothata and De Witt (2020) confirm that the role of occupational therapist is justified in work practice and that occupational therapist is able to offer vocational rehabilitation services. However, with limited published evidence, little is known about occupational therapists’ perceived ability to facilitate reasonable accommodations in the workplace for employees with MDD. The aim of this research was to explore and describe occupational therapists’ perceived ability to facilitate reasonable accommodations in the workplace for employees with MDD.

## Research methods and design

### Study design

A qualitative research approach using an explorative descriptive design (Brink, Van der Walt & Van Rensburg 2018; Grove, Burns & Gray 2013) was employed to explore the perceptions of occupational therapists in facilitating reasonable accommodations for employees with MDD. It allowed the authors to gain insights into reasonable accommodations (Babbie 2013; Colorafi & Evans 2016; Kreuger & Neuman 2006). One-on-one semi-structured interviews were conducted with the occupational therapists, enabling participants to express understanding of their experiences during facilitation of the reasonable accommodation (Creswell 2014; Terre Blanche, Durrheim & Painter 2006).

### Setting

The study focused on occupational therapists working in private practice in South Africa, who treated employees diagnosed with MDD in an acute setting. Employees are employed in competitive employment in various positions.

### Study population and sampling strategy

The population of this study, which was approximately 450, included occupational therapists who: (1) were registered with the Health Professional Council of South Africa as independent occupational therapists; (2) had a minimum of 5 years of experience; (3) were members of the Occupational Therapist Association of South Africa; and (4) worked in mental health and/or vocational rehabilitation, facilitating return-to-work and reasonable accommodation, in the private sector for at least 3 years. The participants excluded from this study were occupational therapists who: (1) worked in academia; and (2) worked in government settings or the public health care sector. Purposive and snowball sampling were used to recruit the participants (Babbie 2013). A homogeneous purposive sample of seven occupational therapists was selected, after recruitment using social media platforms of OTASA and other related WhatsApp professional occupational therapy groups.

Participant demographics are described in Table 1.

**TABLE 1:** Participants’ demographic data.

Participant	Age (years)	Years of experience as occupational therapists	Years of experience in mental health and vocational rehabilitation	Qualifications
1	34	11–15	6–10	BOccTher, Postgrad. Diploma-dual diagnosis
2	57	31–35	31+	BOccTher, Postgrad. Diploma in Voc Rehab, MOccTher
3	52	26–30	11–15	BOccTher, Postgrad. Diploma in Voc Rehab
4	57	31–35	26–30	BOccTher, MOccTher, PhD
5	38	16–20	6–10	BOccTher, Postgrad. Diploma in Voc Rehab
6	56	31–35	31+	BOccTher, MOccTher, PhD
7	39	16–20	11–15	BOccTher, Postgrad. Diploma in Voc Rehab

BOccTher, Bachelor Occupational Therapy; Postgrad., postgraduate; MOccTher, Master of Occupational Therap; Voc Rehab, vocational rehabilitation; PhD, Doctor of Philosophy.

### Data collection

Data were collected during the coronavirus disease 2019 (COVID-19) pandemic in 2020. At this time, occupational therapists were providing emergency services to some clients, while others were working fully online. Potential participants who responded to the request to participate in the research and who met the inclusion criteria were contacted by telephone. A semi-structured interview was used (Polit & Beck 2018) to obtain a detailed description of participants’ accounts of reasonable accommodations. Each one-on-one interview was conducted virtually on Google Meet. It lasted between 60 min and 90 min. Open-ended questions were used during the interview, allowing participants to share their experiences about their perceptions and the facilitation of reasonable accommodations for employees with MDD. Some of the questions that were part of the interview guide are: *From your experience, how do you ensure that your facilitation of reasonable accommodations becomes a success? Which process do you follow when facilitating reasonable accommodations for employees with MDD? What do you think is needed to improve the knowledge of OTs during facilitation of reasonable accommodations for employees with MDD?*

The length of the interview ensured extended engagement with each participant on the topic of reasonable accommodation for employees with MDD. Data collection ceased at participant seven once data saturation was achieved as there was no new information coming. The interview guide was developed with a list of questions guided by person environment occupation participation (PEOP) model. Probing questions were employed to clarify and improve the quality of the data collected. One interview was conducted for the pilot study. A participant for pilot study works extensively in vocational rehabilitation. It was piloted, both theoretically and practically (Buys et al. 2022), which allowed for refinement of some of the questions.

### Measures to ensure trustworthiness

Trustworthiness was pioneered by Lincoln and Guba to establish validity and reliability in qualitative research (Polit & Beck 2018). In this study, validity and reliability were achieved through triangulation, confirmability and member checking.

Methodological triangulation was achieved through using data from the individual interviews and literature to develop a comprehensive understanding of the phenomenon (Polit & Beck 2018). Two independent coders received anonymised transcripts, which they analysed, created codes from and put the codes that were similar together to form themes. They met with the research team to discuss the themes developed, and consensus was reached. This ensured confirmability (Polit & Beck 2018). Accuracy of the findings was achieved through member checking (Polit & Beck 2018). The final themes were sent to the participants to verify that the results accurately reflected the information they had provided (Polit & Beck 2018). After discussion with the research team, these changes were included in the findings.

### Data analysis

Before data analysis commenced, the interview recordings were downloaded onto a password-protected drive. These were electronically transmitted confidentially to an independent, experienced transcriber who signed a confidentiality agreement. These were transcribed verbatim and then returned. Transcripts were verified by listening to the recordings. Listening to the recordings and reading the transcribed data multiple times provided familiarity with the information, allowing for reflection on the overall meaning while making notes in the margins of the transcripts (Botma et al. 2010; Creswell 2014). Inductive thematic analysis to develop themes was employed (Botma et al. 2010; Creswell 2014) A bottom-up approach was used (Creswell & Plano Clark 2011) when analysing data collected from the individual interviews. Refined codes were created by combining initial codes to form themes.

Confidentiality was maintained during data analysis as participants were assigned pseudonyms.

### Ethical considerations

Ethical clearance to conduct this study was obtained from the University of Pretoria Faculty of Health Sciences Research Ethics Committee (No. 444/2020). The participants received full disclosure about the nature of the study through an information leaflet emailed to the participants. In this leaflet, the nature of the study, the duration of the interview (60–90 min), the voluntary nature of their participation and that the study would be conducted online (Botma et al. 2010; De Vos et al. 2011) were described. Participants were made aware that they could withdraw at any point during the study if they wished to do so (Botma et al. 2010; De Vos et al. 2011). Informed consent forms were emailed to the participants to confirm their willingness to participate. Before participating and signing the informed consent, participants were allowed to ask questions to the researcher.

## Results

Two themes emerged from participants’ views to support reasonable workplace accommodations for employees with MDD. The two themes identified were: (1) reasonable accommodations – a dynamic process; and (2) occupational therapy competencies.

### Theme 1: Reasonable accommodation – A dynamic process

According to the participants, reasonable accommodation should follow a process. They described this process as dynamic as it is not rigid and depends on the response of the employee with MDD to ongoing treatment, emphasising that to ensure successful reasonable accommodation, this dynamic process must be followed. Two participants echoed this sentiment, stating that:

‘That it is a dynamic process [*of reasonable accommodation*] and things do change on the way.’ (Participant 3)

‘… you need to have an analytical mind and you also need to understand that the process is dynamic.’ (Participant 5)

**FIGURE 1:**
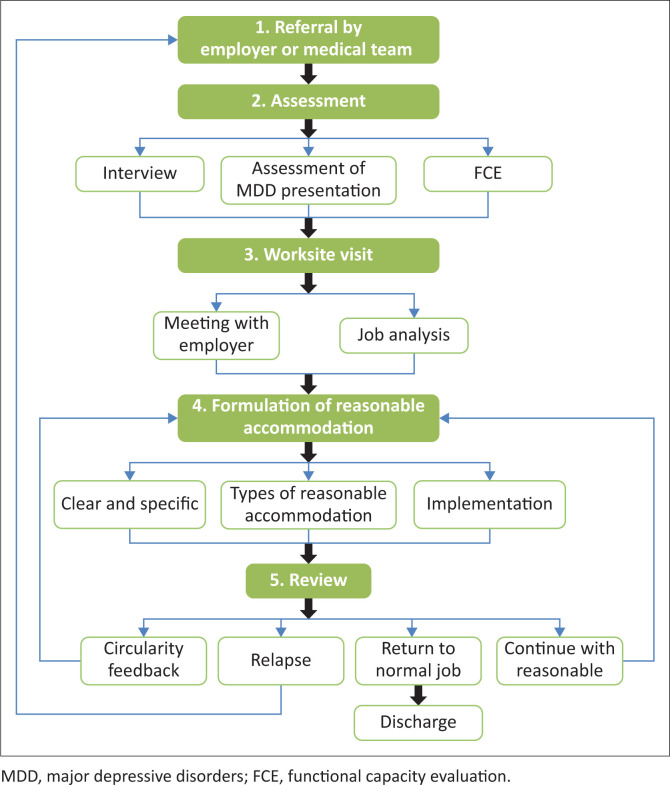
Dynamic process of reasonable accommodation.

This dynamic process (Figure 1) involves five steps identified by the participants as: (1) referral; (2) assessment; (3) worksite visit; (4) formulation of reasonable accommodation; and (5) review.

#### Referral

The initial step of reasonable accommodation begins with a referral from the treating medical professional, insurer or employer who provided information about the pathology. This was supported by one participant who said:

‘I suppose you need to get information from the referring person, I mean psychiatrist or employer. So that’s the referral …’ (Participant 1).

Healthcare professionals treating employees diagnosed with MDD will refer them to an occupational therapist to facilitate the return-to-work process. In some cases, where the employer or insurer struggles to manage an employee’s incapacity, they may refer the employee to an occupational therapist for further management or assistance with reasonable accommodations.

#### Assessment

The second step is an FCE, which includes interviews and employees’ clinical presentation of MDD. These were reported by a participant who remarked:

‘… we have to do a good assessment in the beginning, because everybody’s going to present differently, and it depends on the job’ (Participant 3).

Another participant elaborated that comparing job requirements with employees’ work capacity is essential, as this is part of a functional capacity assessment. This is supported by a participant’s sentiments that:

‘the most important thing is to first assess against the job requirements and the person’s work capacity’ (Participant 2).

Other participants highlighted the need for an occupational therapist to:

‘… sit down and interview the patient [with MDD].’ (Participant 4)

The majority of the participants asserted that assessment of the employees with MDD is vital to determine the presence of clinical symptoms of MDD, such as mood problems, cognitive impairments and functional limitations. It is essential to understand the severity of MDD symptoms. This is substantiated by a participant who remarked:

‘I think what we see the most is the cognitive impact of depression … but also, they are emotional, distracted, so they’re actually not present in the workplace … they’re seeing a drop in performance … we see a lot of absenteeism, leaving work early. So, it’s kind of a combination of absenteeism and presenteeism … we find that there is also relationship breakdown.’ (Participant 1)

Participants were in agreement that the occupational therapist should conduct a full FCE to determine whether the employee with MDD can return to work. This was expressed by one participant who said:

‘[*employers want*] to see if they can get the person [*employee with MDD*] back to work. So, I [*occupational therapist*] will do the full FCE in that case.’ (Participant 7)

#### Worksite visit

With regard to the third step, the participants emphasised the importance of having a clear understanding of the employees’ job roles and engaging in discussions with the employer about the nature of the job and its requirements. This can be achieved through a meeting with an employer representative and conducting a job analysis. The participants also highlighted the necessity of a worksite visit, as they agreed that:

‘The next step would be a work visit, where I [*an occupational therapist*] actually go and have a look at what the employee does [*nature of the job*]. How does it work? Talking to the employer, making contact with the colleagues [*is necessary*].’ (Participant 4)

#### Formulation of reasonable accommodations

Following the fourth step, once the comprehensive assessment is completed, the participants stated that an occupational therapist will need to develop appropriate, reasonable accommodations. The participants advised that the occupational therapist should create reasonable accommodations that are clear and specific, including the types of reasonable accommodations needed and their implementation. They agreed that the occupational therapist must provide relevant and reasonable accommodations. One participant supported this by stating that:

‘… it’s obviously looking at everything, formulating what you need to do, and then provide recommendations.’ (Participant 1)

The participants reported that the facilitation of reasonable accommodations for employees living with MDD should be person specific and not general, given the uniqueness of each employee and their workplace. The participants further highlighted that clear and specific reasonable accommodations were necessary for an employee with MDD, as captured by a participant who said:

‘… we can’t just have a blanket idea of accommodations for person with depression, it doesn’t work.’ (Participant 3)

Some participants provided examples of reasonable accommodations for employees with MDD, including acquiring new equipment and/or adapting the environment, restructuring job functions, a graded return to work, adjusting working time and leave, allowing time off for treatment and providing specialised supervision, training and support. The participants expressed concern that, although the formulated reasonable accommodations could be clear and specific, the implementation thereof might remain a challenge. Implementation of the recommended reasonable accommodations should be ensured by an occupational therapist. This view was articulated by one participant who remarked:

‘… implementation … that’s something that I sometimes have to drive’ (Participant 5).

Another participant emphasised the importance of setting a reasonable duration for accommodations. Where the reasonable accommodation lasts longer than a year, permanent reasonable accommodations should be recommended. This was emphasised by a participant who said:

‘For temporary accommodation, we look at 3 to 6 months. We do get some that go up to a year, but if we’re heading up to a point of the year, then … this should be permanent.’ (Participant 1)

#### Review

The review is the final stage in the process of reasonable accommodation. It concentrates on ongoing feedback, the employee’s return to normal work or the continuation of reasonable accommodations or relapse. Participants emphasised the importance of the review throughout the process, noting that it can take various forms. They advised that feedback from the occupational therapist to the employer is crucial for identifying any additional reasonable accommodations needed to enable the employee to perform their essential duties. The employer must update the occupational therapist on the success or difficulties of the arrangements put in place. If the suggested reasonable accommodations fail, the process may need to restart. Regular follow-up by the occupational therapist with both the employee and employer regarding progress may be necessary. This sentiment was expressed by a participant saying:

‘So, what I normally have is regular sort of, once a week, where I do a telephonic follow-up with client. We’re going to try it for so long, we’ll have meetings, we’ll follow-up so long, so that they know that it’s not an indefinite thing.’ (Participant 2)

The success of reasonable accommodations can be secured through follow-up and feedback, as emphasised by participants. One participant concurred on the importance of follow-up with the utterance:

‘Follow up. For me, follow-up is crucial. You can have the best assessment and the most amazing placement with visit relationship with a patient, but if you don’t follow it up, there’s going to happen something along the line that’s going to cause an issue. That’s going to make this whole thing sink, yeah.’ (Participant 4)

The success of reasonable accommodation is measured by returning employees to their full duties. Participants substantiated this notion when one of them commented:

‘… really making sure that they return to work is a success’ (Participant 5).

If an employee fails to return to normal duties, it indicates to the occupational therapist that an assessment is needed to determine whether further reasonable accommodation is necessary. The participants warned about the possibility of disappointment due to relapse. They reported that employees with MDD may relapse, which could mean restarting the process from the beginning. This concern was voiced by a participant who said:

‘We were going forward and now we have to go back. And I think when it comes to people [*employees*] with MDD relapses can happen’ (Participant 5).

### Theme 2: Occupational therapists’ competencies

In the pursuit to ensure the success of reasonable accommodations, necessary occupational therapists’ competencies should include: (1) staying on the cutting edge; (2) communicating skills; and (3) managing the case.

#### Staying on the cutting edge

It is crucial that occupational therapists remain informed about reasonable accommodations through sufficient knowledge of occupational therapy and capacity building, as advised by participants. Furthermore, occupational therapists require a thorough understanding of occupational therapy principles, including the application of the PEOP model, relevant labour and disability equity laws, and insights into the world of work and the context in which clients operate. This view was supported by a participant who remarked:

‘The PEOP Model. Understanding the environment, the person and the occupations, and the link between all of those things, and how we address the gaps … that’s often how us OTs, and it’s a wonderful thing, where we’re thinking and we’re prioritising our patient, but an OT does need to understand the world of work. You do need to understand productivity.’ (Participant 2)

The other participants echoed their understanding of the MDD pathology in employees, the level of functioning of employees with MDD and the prognosis related to the condition. The participants indicated that mentoring, advanced training, postgraduate diplomas in vocational rehabilitation, ongoing professional development courses (CPD) forums, webinars and interest groups will enhance occupational therapists’ knowledge. They also noted that occupational therapists might utilise academic resources such as journal articles and the Job Accommodation Network as part of capacity building. This view is supported by a participant who said:

‘They can go on vocational rehabilitation, they can learn it, they can work it with other colleagues under mentoring situations or some sort of advanced training’ (Participant 4).

#### Communicating skills

The participants found that essential skills, such as assertiveness, negotiation, clinical reasoning, listening, interviewing and facilitation, are crucial for occupational therapists who recommend reasonable accommodations. They believed that occupational therapists with clinical experience possess advanced clinical reasoning skills necessary for assisting employees with MDD in obtaining suitable accommodations. This is borne out by a participant who said:

‘Your clinical reasoning, your understanding of a case and your ability to formulate what needs to go [*is essential*]’ (Participant 1).

The participants reported that facilitation skills are necessary for occupational therapists to manage employers and other stakeholders. This is supported by a participant who cited:

‘… facilitation skills, in terms of dealing with the employer and all the different stakeholders’ as critical (Participant 3).

#### Managing the case

They further emphasised the importance of managing cases of employees with MDD. During case management, the occupational therapist should manage the expectations of both employee and employer and play the role of a proactive consultant. The significance of case management is supported by a participant who commented:

‘… when you’ve got a clinician and the person [*employee with MDD*] and their insurance company, you [*occupational therapist*] have to be a case manager.’ (Participant 1)

The participants advised that the employees’ MDD may require proper management to prevent it from backfiring. They also reported that all stakeholders should ensure expectations are clearly managed. This view was expressed by the participants who commented:

‘But the thing is, it has to be properly managed [*manage expectations*], otherwise, it can backfire.’ (Participant 2)

The participants emphasised that during consultations with employees suffering from MDD and other stakeholders, occupational therapists must regularly follow-up on the client. This is supported by the participant who said:

‘So, what I normally have is regular sort of, once a week, where I do a telephonic follow-up with client’ (Participant 2).

## Discussion

The study aimed to explore and describe the perceived ability of occupational therapists to facilitate reasonable accommodations in the workplace for employees with MDD. The results indicated that following a dynamic process during facilitation of reasonable accommodation contributes positively towards the ability of occupational therapists. Possessing essential competencies such as staying on the cutting edge, skills and case management further enhances occupational therapist’s ability to facilitate reasonable accommodation

### Reasonable accommodations – A dynamic process

Reasonable accommodation is a dynamic process, and steps included are referral, assessment, worksite visit, formulation of reasonable accommodation, circulatory feedback and review. This is in line with what occupational therapy practice framework 4th edition (OTPF-4), which outlines the occupational therapy process as client centred, occupation based and dynamic. Occupational therapy process includes evaluation, intervention and outcomes (AOTA 2020).

#### Referral

Referral is the initial step in the reasonable accommodation process. Swart and Buys (2014) advised that service delivery and payment boundaries should be clearly indicated in the referral. Moreover, the referral letter must include comprehensive details from medical reports, job descriptions and the employee’s work performance (Buys 2015). Providing comprehensive and detailed information in the referral letter can facilitate the preparation for the interview and assessment of the specific employee.

#### Assessment

Assessment should concentrate on ‘finding out what the client wants and needs to do; determining what the client can do and has done; and identifying supports and barriers to health, well-being, and participation’ (AOTA 2020). Assessments in the form of FCE should include an interview, evaluation of MDD clinical presentation and aspects of the occupational therapy domain. Swart et al. (2024) add that an interview guide covering the client’s educational background, work history, psychiatric history, other medical history, current treatment and functional status should be developed before the occupational therapist contacts the employee. During the interview, an occupational therapist must observe and identify MDD symptoms such as emotional problems, cognitive impairments and functional limitations. The selection of appropriate assessment tools to perform an FCE should be guided by the referral letter, interview and observations of MDD symptoms. The findings indicated that FCE aids occupational therapists in making return-to-work decisions and identifying potential reasonable accommodations to recommend.

#### Worksite visit

Worksite visits are important during the reasonable accommodation process as it provides an opportunity for the occupational therapists to meet with the employer and conduct a job analysis. This study suggests that a job analysis can provide occupational therapists with information about job demands and insight into the workplace culture. Worksite visits help to understand possible reasonable accommodations and positions that may need realignment, as well as to acquire a comprehensive view of the job description (Swart et al. 2024). In South Africa, EEA (1998) makes provision for reasonable accommodation. *Promotion of Equality and Prevention of Unfair Discrimination Act* (PEPUDA) (2000) states that reasonably accommodating people with disabilities will ensure their social transformation. *Promotion of Equality and Prevention of Unfair Discrimination Act* (2000) provides recourse for refusal of reasonable accommodation under EEA (1998). Ramano and Buys (2018) claim that the results of FCE may help in developing modifications to the employee’s workstation or workplace. Occupational therapists can negotiate and implement reasonable accommodations for individuals with MDD. Importantly, occupational therapists should ensure that they recommend clear and specific accommodations tailored to each employer and their specific job, as this approach will enhance the successful implementation of these accommodations.

#### Formulation of reasonable accommodation

Generic reasonable accommodations are ineffective; therefore, it is prudent to tailor clear and specific reasonable accommodations that are individualised to each employee and their particular job. This aligns with Gold et al. (2012), who indicated that developed reasonable accommodations, firmly matched to employees’ specific job functions, will assist them in justifying their case. The ambiguity of the reasonable accommodation negotiation makes it difficult for the employer and employee to provide and receive reasonable accommodation (Hossain et al. 2021). Rangarajan et al. (2020) who focused on workplace reasonable accommodation for professionals with severe mental illness, warned that reasonable accommodation is not ‘one size fits all’ but should be tailored to the individual. Stergiou-Kita et al. (2016) further established that vague reasonable accommodations are ineffective and are seen as a ‘want’ rather than a ‘reasonable and justifiable need’. Types of reasonable accommodations for employees with MDD are listed in Table 2 as proposed by the participants of this study.

**TABLE 2:** Types of reasonable accommodation.

Types of reasonable accommodation	Examples
1. Graded return-to-work	Half-a-day to full day Few days a week
2. Restructuring job functions	Reduced targets Reduced functions or tasks Removing non-essential tasks
3. Adjusting working hours	Flexi hours Adjusting time to report for duty in the morning Change shift hours to office hours
4. Allowing time off for treatment	Appointments with the treating team Collection of medication
5. Providing specialised equipment	Noise cancellation headsets
6. Training and support in the workplace	Support from the supervisor

Bastien and Corbière (2019), Bolo et al. (2013), McDowell and Fossey (2015) and Rangarajan et al. (2020) agree on reasonable accommodations listed in Table 2, such as flexible work schedules, reduced hours, modified training and supervision, task modifications, job changes or altered job duties or descriptions, environmental adjustments or physical accommodations and reduced work hours. However, the study by McDowell and Fossey (2015) highlights that from the employee’s perspective, changes to work schedules are the most difficult accommodations to negotiate. Providing reasonable accommodation does not only benefit the employee but the company likewise, as it ensures work productivity (MacDonald-Wilson, Fabian & Dong 2025).

#### Review

The review is significant, and it completes the dynamic process of reasonableness. Furthermore, the review encompasses circulatory follow-up feedback, the return to normal work, the continuation of reasonable accommodation and relapse. The review of occupational therapy interventions and services is instrumental in ensuring quality and maintaining relevance to their intended purpose. Changes in the intervention plan are guided by a continuous process of review and re-evaluation. The review steps involve reassessing the plan and its implementation in terms of achieving outcomes, making necessary adjustments and deciding whether to continue, discharge or refer to other practitioners. Occupational therapists should provide feedback to the employer regarding the reasonable accommodations that are to be implemented, while the employer must also provide feedback to the occupational therapist on the employee’s performance, including successes and challenges with the reasonable accommodations. The process may need to restart if any difficulties are encountered during the implementation of reasonable accommodations. Continuous follow-up between the occupational therapist, employer and employee is essential, whether by telephone, in person or via email. Follow-up could be conducted weekly initially, then gradually reduced to monthly as the employee improves. Buys (2015) advises that follow-up should be done with both the employee and employer and that it ‘closes the case’. The importance of ongoing follow-up feedback is to sustain progress and ensure the success of reasonable accommodations. The primary goal of reasonable accommodation is to return employees to their normal work functions. If the employee does not improve, reasonable accommodation continues and necessary adjustments are re-evaluated accordingly. Following re-evaluation, intervention may resume if ongoing service is required, and modifications to the intervention plan may occur. Occupational therapists should note that some employees may relapse, requiring the process of reasonable accommodations (Figure 1) to be restarted. Schlicker et al. (2018) add that there is a possibility of relapse and recurrence even after treatment is completed. Challenges at work may trigger a relapse of depression.

### Occupational therapist competencies

Staying at the cutting edge, communicating skills and managing the case are essential competencies that occupational therapists must possess when facilitating reasonable accommodations to ensure their success. Buys (2015) highlighted knowledge, skills and values as prerequisites for delivering vocational rehabilitation services (Buys 2015). Knowledge, experience and skills were identified by Ramano and Buys (2018) as important competencies required for occupational therapists to carry out an FCE. Ramano and Buys (2018) emphasised that the competency of knowledge includes understanding pathology, the occupational therapy process, occupational theories, assessment, economic climate, legislation and the world of work. The study also found that occupational therapists’ knowledge should encompass the application of the PEOP model, legislation and understanding the world of work and the context in which the client operates. Mentoring, postgraduate courses, continuing professional development courses, interest groups, webinars and forums promote capacity building are vital for advancing occupational therapy knowledge. This aligns with the systematic review by DeCorby-Watson et al. (2018), where participants indicated that capacity-building interventions – including training and workshops, internet-based instruction, self-directed learning, communities of practice, technical assistance and multi-strategy approaches – enhance knowledge, skills and self-efficacy. Therefore, occupational therapists should utilise various platforms to continually develop and improve their expertise and knowledge. Experience in the field of work, vocational rehabilitation and mental health, along with skills and solid knowledge, are essential for occupational therapists to make effective return-to-work decisions.

Occupational therapists need to possess clinical reasoning skills, listening skills, assertiveness, interview, negotiation and facilitation skills, which are considered necessary for facilitating reasonable accommodations. These skills enable occupational therapists to negotiate effectively with employers and other stakeholders. Similar findings were reported by Bade and Eckert (2008), Ramano and Buys (2018) and Buys (2015). Bade and Eckert (2008) noted that occupational therapists have a distinctive value grounded in comprehensive training in psychosocial and psychosocial sciences, as well as negotiation skills. Ramano and Buys (2018) highlight advocacy, clinical reasoning, interview, negotiation, assertiveness and communication skills as crucial for occupational therapists. Use of empathy and client centredness during the facilitation of reasonable accommodations is necessary, as these aspects can significantly impact employees’ careers and lives.

Parker et al. (2019) stated that client centredness permits autonomy and allows the employee with disability a choice. Buys (2015) recommended that occupational therapists must understand and implement case management, which is one of the vocational rehabilitation services. Occupational therapists must manage employer and employee expectations during case management to ensure the process does not backfire. Occupational therapists must proactively consult with employees with MDD and other stakeholders. It is vital that case management is carried out regularly to keep the process moving regarding recommended reasonable accommodation.

### Strengths and limitations

The strength of this study lies in its pioneering nature in South Africa, focusing on reasonable accommodation within the occupational therapy profession. The interview guide was well designed, providing data saturation with seven participants. There were limitations to the virtual interviews, which impacted the ability to observe treatment areas in person. The study’s focus was solely on facilitating reasonable accommodation for employees with MDD.

## Conclusion

Occupational therapists being part of the MDD treating team have the ability to facilitate the dynamic process of reasonable accommodations. The study generated a dynamic process of reasonable accommodation that occupational therapists can employ. Furthermore, the findings of this study could serve as guidance for occupational therapists when facilitating reasonable accommodation for employees with MDD. Reasonable accommodation is an essential legal provision that enables employees with MDD to return to work. Effective facilitation of reasonable accommodation requires occupational therapists to stay up to date with the latest knowledge and practice (on the cutting edge), possess relevant skills and manage cases effectively. These results provide valuable insights into the facilitation of reasonable accommodation within the occupational therapy profession, particularly for those practising in mental health, vocational rehabilitation and the treatment of employees with MDD.

### Contribution

This study developed the dynamic process of reasonable accommodation. This process may serve as a guideline for occupational therapists when facilitating reasonable accommodations. The specific reasonable accommodations for employees with MDD outlined in this study can be a resource for occupational therapists in formulating suitable adjustments for their patients diagnosed with MDD. Possible accommodations include acquiring new equipment (e.g. noise-cancelling headphones), environmental adjustments, restructuring job functions (focusing on key responsibilities, graded return-to-work schedules such as working a few days a week or half-days and reducing workload capacity), allowing time off for treatment, providing specialised supervision, adjusting working hours and leave (such as flexi hours or office hours instead of shift hours), as well as training and support. Staying up to date with the latest knowledge and practice, developing professional skills and case management are essential competencies for occupational therapists. They are encouraged to enhance their capabilities through various platforms such as mentoring, continuing professional development courses, interest groups, postgraduate studies, webinars and forums to gain the necessary knowledge and skills in vocational rehabilitation and mental health. This study has emphasised the important role of occupational therapists in the process of reasonable accommodation and recognises them as the key drivers of such adjustments in South Africa. Overall, this research contributes valuable knowledge to the practice of occupational therapy.
